# Bis{2-[(2-benzoyl­hydrazin-1-yl­idene)meth­yl]-6-meth­oxy­phenolato}iron(III) chloride monohydrate

**DOI:** 10.1107/S1600536810023226

**Published:** 2010-06-23

**Authors:** Li-Fei Zou, Yu-Qin Ma, Gui-Miao Yu, Feng-Jiao Gan, Yun-Hui Li

**Affiliations:** a School of Chemistry and Environmental Engineering, Changchun University of Science and Technology, Changchun 130022, People’s Republic of China

## Abstract

In the title mononuclear iron(III) complex, [Fe(C_15_H_13_N_2_O_3_)_2_]Cl·H_2_O, the Fe^III^ atom has a distorted octa­hedral geometry and is six-coordinated by four O atoms and two N atoms from two ligands. In the crystal structure, the complex cations, Cl^−^ anions and water mol­ecules are connected into a chain along [100] through N—H⋯O, O—H⋯Cl and N—H⋯Cl hydrogen bonds. Two adjacent chains are linked by O—H⋯O hydrogen bonds.

## Related literature

For the applications of metal–Schiff base compounds, see: Dilworth (1976 ▶); Merchant & Clothia (1970 ▶); Pickart *et al.* (1983 ▶). For the ligand synthesis, see: Pouralimardan *et al.* (2007 ▶); Sacconi (1954 ▶). For related structures, see: Gao *et al.* (1998 ▶); Monfared *et al.* (2007 ▶); Yu *et al.* (2010 ▶).
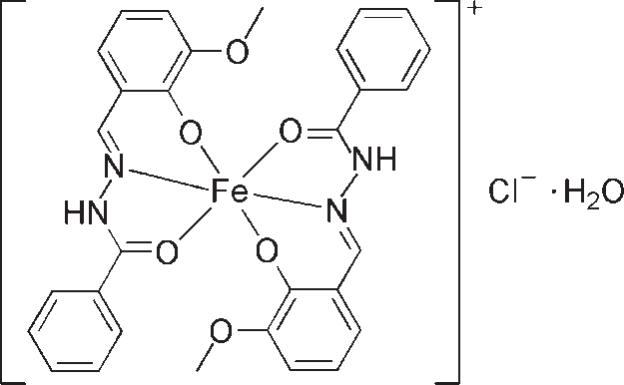


## Experimental

### 

#### Crystal data

[Fe(C_15_H_13_N_2_O_3_)_2_]Cl·H_2_O
*M* *_r_* = 647.86
Monoclinic, 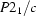

*a* = 12.7778 (10) Å
*b* = 22.7113 (18) Å
*c* = 10.0604 (7) Å
β = 94.542 (1)°
*V* = 2910.4 (4) Å^3^
*Z* = 4
Mo *K*α radiation
μ = 0.67 mm^−1^
*T* = 296 K
0.24 × 0.18 × 0.15 mm

#### Data collection

Bruker SMART APEX CCD diffractometer
Absorption correction: multi-scan (*SADABS*; Sheldrick, 1996 ▶) *T*_min_ = 0.857, *T*_max_ = 0.907
14540 measured reflections
5098 independent reflections
3508 reflections with *I* > 2σ(*I*)
*R*_int_ = 0.052

#### Refinement

*R*[*F* ^2^ > 2σ(*F* ^2^)] = 0.051
*wR*(*F* ^2^) = 0.153
*S* = 0.98
5098 reflections
390 parameters
H-atom parameters constrained
Δρ_max_ = 0.95 e Å^−3^
Δρ_min_ = −0.48 e Å^−3^

### 

Data collection: *SMART* (Bruker, 2007 ▶); cell refinement: *SAINT-Plus* (Bruker, 2007 ▶); data reduction: *SAINT-Plus*; program(s) used to solve structure: *SHELXS97* (Sheldrick, 2008 ▶); program(s) used to refine structure: *SHELXL97* (Sheldrick, 2008 ▶); molecular graphics: *DIAMOND* (Brandenburg, 1999 ▶); software used to prepare material for publication: *SHELXL97* and *publCIF* (Westrip, 2010 ▶).

## Supplementary Material

Crystal structure: contains datablocks I, global. DOI: 10.1107/S1600536810023226/hy2322sup1.cif

Structure factors: contains datablocks I. DOI: 10.1107/S1600536810023226/hy2322Isup2.hkl

Additional supplementary materials:  crystallographic information; 3D view; checkCIF report

## Figures and Tables

**Table 1:** Selected bond lengths (Å)

Fe1—O1	2.070 (3)
Fe1—O2	1.904 (3)
Fe1—O4	2.062 (3)
Fe1—O5	1.901 (3)
Fe1—N2	2.106 (3)
Fe1—N4	2.124 (3)

**Table 2:** Hydrogen-bond geometry (Å, °)

*D*—H⋯*A*	*D*—H	H⋯*A*	*D*⋯*A*	*D*—H⋯*A*
N1—H1*B*⋯Cl1^i^	0.86	2.25	3.087 (3)	163
N3—H3*B*⋯O1*W*	0.86	1.92	2.759 (4)	164
O1*W*—H1*WA*⋯O5^ii^	0.85	2.39	3.045 (4)	134
O1*W*—H1*WB*⋯Cl1	0.85	2.37	3.198 (3)	163

Symmetry codes: (i) 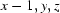; (ii) 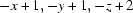.

**Fig. 1.**
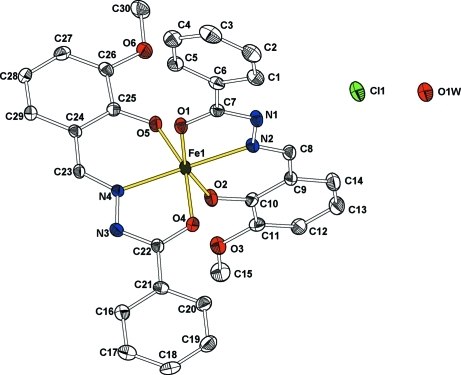
Molecular structure of the title compound. H atoms are omitted for clarity. Displacement ellipsoids are drawn at the 30% probability level.

**Fig. 2.**
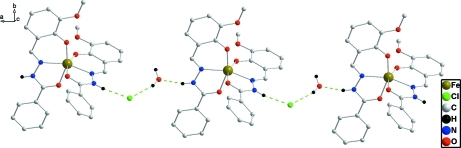
One-dimensional chain structure in the title compound. Hydrogen bonds are shown as green dashed lines.

## supplementary crystallographic information

### Comment

Studies of acylhydrazone Schiff base and the dependence of their chelation mode with transition metal ions have been of significant interest. On one hand, their metal compounds have been reported to act as enzyme inhibitors (Dilworth, 1976) and are useful due to their pharmacological applications (Merchant & Clothia, 1970). On the other hand, it seems to be a good candidate for catalytic oxidation studies because of their stability to resist oxidation (Pickart *et al.*, 1983). These findings have triggered the exploration of new molecular clusters based on acylhydrazone Schiff base. During the last several years, the crystal structures of metal compounds with 3-methoxysalicylaldehyde benzoylhydrazide have been attracted tremendous interest (Gao *et al.*, 1998; Monfared *et al.*, 2007; Yu *et al.*, 2010). As a continuation of our effort in this system, the preparation and crystal structure of the title Schiff base iron(III) compound are reported here.

The molecular structure of the title compound is illustrated in Fig. 1, which consists of one mononuclear [Fe(C_15_H_13_N_2_O_3_)_2_]^+^ cation, one Cl^-^ anion and one water molecule. The Fe^III^ atom has a distorted octahedral geometry and is six-coordinated by four O atoms and two N atoms from two ligands (Table 1). In one ligand, the strained angle of O1—Fe1—N2 [74.54 (11)°] correlates with the bite angle for the five-membered chelate ring Fe1—O1—C7—N1—N2, and the loose angle of O2—Fe1—N2 [84.74 (11)°] correlates with the six-membered ring Fe1—N2—C8—C9—C10—O2. The axial angle N2—Fe1—N4 [159.46 (12)°] deviates significantly from the ideal 180°. Similar case occurrs for another ligand. In the crystal structure, the complex cations, Cl^-^ anions and water molecules are connected into a chain through N—H···O, O—H···Cl and N—H···Cl hydrogen bonds. Two adjacent chains are linked by O—H···O hydrogen bonds. (Fig. 2 and Table 2).

### Experimental

The 3-methoxysalicylaldehyde benzoylhydrazide ligand (H_2_*L*) was prepared in a similar manner according to the reported procedures (Pouralimardan *et al.*, 2007; Sacconi, 1954). The title compound was synthesized by adding FeCl_3_.6H_2_O (27.0 mg, 0.1 mmol) to a solution of H_2_*L* (27.3 mg, 0.10 mmol) in methanol (15 ml). The resulting mixture was stirred for 3 h at room temperature to afford a dark brown solution and then filtered. The filtrate was allowed to stand at room temperature for about three weeks and black crystals were produced at the bottom of the vessel on slow evaporation of methanol.

### Refinement

All H atoms were placed in calculated positions and refined using a riding model, with C—H = 0.93 (aromatic), 0.96 (methyl) Å and N—H = 0.86 Å and with *U*_iso_(H) = 1.2(1.5 for methyl)*U*_eq_(C, N). Water H atoms were located in a difference Fourier map and refined as riding, with O—H = 0.85 Å and *U*_iso_(H) = 1.2*U*_eq_(O).

### Crystal data

**Table:**

[Fe(C_15_H_13_N_2_O_3_)_2_]Cl·H_2_O	*F*(000) = 1340
*M**_r_* = 647.86	*D*_x_ = 1.479 Mg m^−^^3^
Monoclinic, *P*2_1_/*c*	Mo *K*α radiation, λ = 0.71073 Å
Hall symbol: -P 2ybc	Cell parameters from 4767 reflections
*a* = 12.7778 (10) Å	θ = 4.8–51.7°
*b* = 22.7113 (18) Å	µ = 0.67 mm^−^^1^
*c* = 10.0604 (7) Å	*T* = 296 K
β = 94.542 (1)°	Block, black
*V* = 2910.4 (4) Å^3^	0.24 × 0.18 × 0.15 mm
*Z* = 4	

### Data collection

**Table:**

Bruker SMART APEX CCD diffractometer	5098 independent reflections
Radiation source: fine-focus sealed tube	3508 reflections with *I* > 2σ(*I*)
graphite	*R*_int_ = 0.052
φ and ω scans	θ_max_ = 25.0°, θ_min_ = 1.8°
Absorption correction: multi-scan (*SADABS*; Sheldrick, 1996)	*h* = −15→15
*T*_min_ = 0.857, *T*_max_ = 0.907	*k* = −27→23
14540 measured reflections	*l* = −11→10

### Refinement

**Table:**

Refinement on *F*^2^	Primary atom site location: structure-invariant direct methods
Least-squares matrix: full	Secondary atom site location: difference Fourier map
*R*[*F*^2^ > 2σ(*F*^2^)] = 0.051	Hydrogen site location: inferred from neighbouring sites
*wR*(*F*^2^) = 0.153	H-atom parameters constrained
*S* = 0.98	*w* = 1/[σ^2^(*F*_o_^2^) + (0.0933*P*)^2^ + 0.1872*P*] where *P* = (*F*_o_^2^ + 2*F*_c_^2^)/3
5098 reflections	(Δ/σ)_max_ < 0.001
390 parameters	Δρ_max_ = 0.95 e Å^−^^3^
0 restraints	Δρ_min_ = −0.47 e Å^−^^3^

### Fractional atomic coordinates and isotropic or equivalent isotropic displacement parameters (Å^2^)

**Table:**

	*x*	*y*	*z*	*U*_iso_*/*U*_eq_	
Fe1	0.28270 (4)	0.56160 (2)	0.90592 (5)	0.02612 (19)	
Cl1	0.87327 (8)	0.66511 (5)	0.74420 (12)	0.0455 (3)	
C1	0.0861 (3)	0.69236 (18)	0.5203 (4)	0.0376 (10)	
H1A	0.0396	0.7024	0.5832	0.045*	
C2	0.0804 (4)	0.7195 (2)	0.3979 (5)	0.0449 (12)	
H2A	0.0305	0.7487	0.3787	0.054*	
C3	0.1472 (4)	0.7040 (2)	0.3036 (5)	0.0511 (13)	
H3A	0.1416	0.7227	0.2211	0.061*	
C4	0.2219 (4)	0.6615 (2)	0.3292 (4)	0.0433 (11)	
H4A	0.2659	0.6508	0.2640	0.052*	
C5	0.2313 (3)	0.63511 (19)	0.4515 (4)	0.0350 (10)	
H5A	0.2837	0.6073	0.4703	0.042*	
C6	0.1631 (3)	0.64933 (18)	0.5489 (4)	0.0307 (9)	
C7	0.1786 (3)	0.61984 (17)	0.6799 (4)	0.0275 (9)	
C8	0.0525 (3)	0.58652 (18)	0.9627 (4)	0.0310 (9)	
H8A	−0.0129	0.6023	0.9359	0.037*	
C9	0.0651 (3)	0.55879 (18)	1.0885 (4)	0.0294 (9)	
C10	0.1608 (3)	0.53399 (17)	1.1403 (4)	0.0290 (9)	
C11	0.1644 (3)	0.50693 (18)	1.2666 (4)	0.0319 (10)	
C12	0.0756 (3)	0.5034 (2)	1.3356 (4)	0.0397 (11)	
H12A	0.0786	0.4844	1.4177	0.048*	
C13	−0.0175 (3)	0.5278 (2)	1.2842 (4)	0.0440 (12)	
H13A	−0.0766	0.5256	1.3322	0.053*	
C14	−0.0234 (3)	0.5551 (2)	1.1631 (4)	0.0405 (11)	
H14A	−0.0867	0.5716	1.1294	0.049*	
C15	0.2726 (4)	0.4590 (2)	1.4393 (4)	0.0500 (13)	
H15A	0.3446	0.4478	1.4594	0.075*	
H15B	0.2288	0.4246	1.4392	0.075*	
H15C	0.2522	0.4862	1.5056	0.075*	
C16	0.5865 (3)	0.71820 (18)	1.0512 (4)	0.0353 (10)	
H16A	0.6324	0.6914	1.0169	0.042*	
C17	0.6243 (4)	0.76907 (19)	1.1109 (4)	0.0400 (11)	
H17A	0.6961	0.7766	1.1178	0.048*	
C18	0.5563 (4)	0.8090 (2)	1.1605 (4)	0.0435 (12)	
H18A	0.5822	0.8436	1.2003	0.052*	
C19	0.4499 (4)	0.7979 (2)	1.1514 (4)	0.0444 (12)	
H19A	0.4043	0.8250	1.1852	0.053*	
C20	0.4108 (3)	0.74682 (18)	1.0923 (4)	0.0356 (10)	
H20A	0.3389	0.7395	1.0863	0.043*	
C21	0.4784 (3)	0.70675 (17)	1.0423 (4)	0.0294 (9)	
C22	0.4344 (3)	0.65140 (17)	0.9850 (4)	0.0257 (9)	
C23	0.5028 (3)	0.52154 (17)	0.8316 (4)	0.0257 (9)	
H23A	0.5735	0.5297	0.8244	0.031*	
C24	0.4618 (3)	0.46776 (17)	0.7770 (4)	0.0267 (9)	
C25	0.3558 (3)	0.45109 (17)	0.7842 (4)	0.0269 (9)	
C26	0.3226 (3)	0.39603 (18)	0.7277 (4)	0.0303 (9)	
C27	0.3928 (3)	0.36044 (19)	0.6688 (4)	0.0351 (10)	
H27A	0.3703	0.3246	0.6319	0.042*	
C28	0.4968 (3)	0.37752 (19)	0.6638 (4)	0.0366 (10)	
H28A	0.5434	0.3527	0.6246	0.044*	
C29	0.5312 (3)	0.42949 (18)	0.7147 (4)	0.0327 (10)	
H29A	0.6008	0.4404	0.7091	0.039*	
C30	0.1794 (4)	0.3313 (2)	0.6782 (6)	0.0551 (14)	
H30A	0.1063	0.3280	0.6928	0.083*	
H30B	0.2167	0.2981	0.7175	0.083*	
H30C	0.1874	0.3321	0.5842	0.083*	
N1	0.1015 (3)	0.61943 (14)	0.7608 (3)	0.0307 (8)	
H1B	0.0412	0.6352	0.7399	0.037*	
N2	0.1262 (2)	0.59143 (14)	0.8822 (3)	0.0260 (7)	
N3	0.4973 (2)	0.61077 (13)	0.9384 (3)	0.0277 (8)	
H3B	0.5640	0.6158	0.9378	0.033*	
N4	0.4475 (2)	0.55953 (13)	0.8906 (3)	0.0231 (7)	
O1	0.2638 (2)	0.59602 (12)	0.7150 (3)	0.0307 (6)	
O1W	0.7122 (2)	0.60423 (13)	0.9294 (3)	0.0421 (8)	
H1WA	0.7368	0.5719	0.9611	0.050*	
H1WB	0.7434	0.6240	0.8725	0.050*	
O2	0.2468 (2)	0.53545 (13)	1.0763 (3)	0.0346 (7)	
O3	0.2611 (2)	0.48627 (13)	1.3112 (3)	0.0396 (7)	
O4	0.3375 (2)	0.64162 (12)	0.9791 (3)	0.0318 (7)	
O5	0.2873 (2)	0.48311 (12)	0.8409 (3)	0.0323 (7)	
O6	0.2204 (2)	0.38391 (13)	0.7375 (3)	0.0423 (8)	

### Atomic displacement parameters (Å^2^)

**Table:**

	*U*^11^	*U*^22^	*U*^33^	*U*^12^	*U*^13^	*U*^23^
Fe1	0.0196 (3)	0.0345 (3)	0.0245 (3)	0.0041 (2)	0.0039 (2)	0.0024 (3)
Cl1	0.0289 (6)	0.0524 (7)	0.0550 (8)	0.0074 (5)	0.0009 (5)	0.0078 (6)
C1	0.036 (3)	0.040 (3)	0.036 (3)	0.001 (2)	−0.0045 (19)	0.007 (2)
C2	0.045 (3)	0.045 (3)	0.043 (3)	0.001 (2)	−0.008 (2)	0.013 (2)
C3	0.055 (3)	0.062 (3)	0.034 (3)	−0.011 (3)	−0.010 (2)	0.024 (2)
C4	0.046 (3)	0.055 (3)	0.029 (3)	−0.001 (2)	0.001 (2)	0.008 (2)
C5	0.036 (2)	0.042 (3)	0.027 (2)	0.001 (2)	0.0000 (19)	0.002 (2)
C6	0.029 (2)	0.036 (2)	0.027 (2)	−0.0047 (18)	−0.0044 (18)	0.0020 (18)
C7	0.028 (2)	0.028 (2)	0.026 (2)	−0.0035 (17)	0.0018 (17)	−0.0018 (17)
C8	0.026 (2)	0.041 (2)	0.026 (2)	0.0031 (18)	0.0046 (18)	0.0017 (19)
C9	0.024 (2)	0.038 (2)	0.026 (2)	0.0022 (18)	0.0032 (17)	−0.0015 (19)
C10	0.028 (2)	0.032 (2)	0.028 (2)	0.0005 (17)	0.0064 (17)	−0.0040 (18)
C11	0.036 (2)	0.035 (2)	0.025 (2)	−0.0016 (19)	0.0011 (18)	−0.0016 (19)
C12	0.037 (3)	0.056 (3)	0.026 (2)	−0.003 (2)	0.0081 (19)	0.001 (2)
C13	0.034 (3)	0.068 (3)	0.032 (3)	−0.002 (2)	0.013 (2)	0.005 (2)
C14	0.030 (2)	0.058 (3)	0.034 (3)	0.006 (2)	0.0046 (19)	0.007 (2)
C15	0.056 (3)	0.068 (3)	0.026 (2)	0.006 (3)	0.003 (2)	0.017 (2)
C16	0.033 (2)	0.036 (2)	0.037 (3)	0.0015 (19)	0.0023 (19)	−0.001 (2)
C17	0.043 (3)	0.039 (3)	0.036 (3)	−0.005 (2)	−0.005 (2)	0.002 (2)
C18	0.062 (3)	0.039 (3)	0.028 (2)	−0.006 (2)	−0.003 (2)	−0.009 (2)
C19	0.052 (3)	0.041 (3)	0.040 (3)	0.010 (2)	0.003 (2)	−0.009 (2)
C20	0.040 (3)	0.038 (2)	0.029 (2)	0.006 (2)	0.0011 (19)	−0.0023 (19)
C21	0.032 (2)	0.033 (2)	0.022 (2)	−0.0003 (18)	−0.0045 (17)	0.0024 (18)
C22	0.023 (2)	0.033 (2)	0.021 (2)	0.0052 (17)	−0.0003 (16)	0.0050 (17)
C23	0.020 (2)	0.037 (2)	0.020 (2)	0.0047 (17)	0.0027 (16)	0.0028 (17)
C24	0.022 (2)	0.037 (2)	0.020 (2)	0.0021 (17)	−0.0017 (16)	0.0021 (18)
C25	0.028 (2)	0.033 (2)	0.019 (2)	0.0039 (17)	0.0007 (17)	0.0034 (17)
C26	0.027 (2)	0.037 (2)	0.027 (2)	−0.0001 (18)	−0.0022 (17)	0.0054 (18)
C27	0.043 (3)	0.035 (2)	0.027 (2)	0.002 (2)	−0.0001 (19)	−0.0036 (19)
C28	0.033 (2)	0.043 (3)	0.034 (2)	0.008 (2)	0.0037 (19)	−0.010 (2)
C29	0.025 (2)	0.041 (3)	0.032 (2)	0.0027 (18)	0.0034 (18)	−0.0056 (19)
C30	0.041 (3)	0.047 (3)	0.077 (4)	−0.013 (2)	−0.001 (3)	−0.012 (3)
N1	0.0245 (18)	0.040 (2)	0.0274 (19)	0.0083 (15)	0.0023 (14)	0.0098 (15)
N2	0.0231 (17)	0.0344 (19)	0.0201 (17)	0.0038 (14)	−0.0001 (14)	0.0063 (14)
N3	0.0231 (17)	0.0315 (18)	0.0281 (19)	−0.0025 (14)	0.0002 (14)	−0.0003 (15)
N4	0.0208 (16)	0.0286 (17)	0.0197 (16)	0.0011 (14)	−0.0008 (13)	−0.0002 (14)
O1	0.0250 (15)	0.0424 (17)	0.0251 (15)	0.0053 (13)	0.0053 (12)	0.0069 (13)
O1W	0.0332 (17)	0.0456 (19)	0.0476 (19)	0.0050 (14)	0.0044 (14)	0.0046 (15)
O2	0.0249 (15)	0.0553 (19)	0.0242 (15)	0.0088 (13)	0.0043 (12)	0.0116 (13)
O3	0.0346 (17)	0.059 (2)	0.0248 (16)	0.0076 (15)	0.0022 (13)	0.0145 (14)
O4	0.0279 (16)	0.0359 (16)	0.0318 (16)	0.0046 (12)	0.0033 (12)	−0.0048 (13)
O5	0.0235 (15)	0.0342 (16)	0.0399 (17)	0.0001 (12)	0.0073 (12)	−0.0046 (13)
O6	0.0303 (17)	0.0440 (18)	0.053 (2)	−0.0081 (14)	0.0050 (14)	−0.0108 (15)

### Geometric parameters (Å, °)

**Table:**

Fe1—O1	2.070 (3)	C16—C17	1.372 (6)
Fe1—O2	1.904 (3)	C16—C21	1.402 (6)
Fe1—O4	2.062 (3)	C16—H16A	0.9300
Fe1—O5	1.901 (3)	C17—C18	1.377 (6)
Fe1—N2	2.106 (3)	C17—H17A	0.9300
Fe1—N4	2.124 (3)	C18—C19	1.379 (7)
C1—C2	1.373 (6)	C18—H18A	0.9300
C1—C6	1.401 (6)	C19—C20	1.379 (6)
C1—H1A	0.9300	C19—H19A	0.9300
C2—C3	1.372 (7)	C20—C21	1.377 (5)
C2—H2A	0.9300	C20—H20A	0.9300
C3—C4	1.367 (6)	C21—C22	1.476 (5)
C3—H3A	0.9300	C22—O4	1.254 (4)
C4—C5	1.365 (6)	C22—N3	1.334 (5)
C4—H4A	0.9300	C23—N4	1.289 (5)
C5—C6	1.399 (6)	C23—C24	1.422 (5)
C5—H5A	0.9300	C23—H23A	0.9300
C6—C7	1.478 (5)	C24—C25	1.413 (5)
C7—O1	1.241 (5)	C24—C29	1.422 (5)
C7—N1	1.328 (5)	C25—O5	1.303 (4)
C8—N2	1.294 (4)	C25—C26	1.425 (5)
C8—C9	1.411 (5)	C26—O6	1.346 (5)
C8—H8A	0.9300	C26—C27	1.375 (6)
C9—C10	1.408 (5)	C27—C28	1.389 (6)
C9—C14	1.409 (5)	C27—H27A	0.9300
C10—O2	1.317 (4)	C28—C29	1.347 (6)
C10—C11	1.409 (5)	C28—H28A	0.9300
C11—O3	1.364 (5)	C29—H29A	0.9300
C11—C12	1.379 (5)	C30—O6	1.417 (5)
C12—C13	1.375 (6)	C30—H30A	0.9600
C12—H12A	0.9300	C30—H30B	0.9600
C13—C14	1.364 (6)	C30—H30C	0.9600
C13—H13A	0.9300	N1—N2	1.391 (4)
C14—H14A	0.9300	N1—H1B	0.8600
C15—O3	1.428 (5)	N3—N4	1.394 (4)
C15—H15A	0.9600	N3—H3B	0.8600
C15—H15B	0.9600	O1W—H1WA	0.8500
C15—H15C	0.9600	O1W—H1WB	0.8502
			
O5—Fe1—O2	91.94 (12)	C21—C16—H16A	120.1
O5—Fe1—O4	158.43 (11)	C16—C17—C18	120.2 (4)
O2—Fe1—O4	93.00 (12)	C16—C17—H17A	119.9
O5—Fe1—O1	92.26 (11)	C18—C17—H17A	119.9
O2—Fe1—O1	159.12 (11)	C17—C18—C19	120.2 (4)
O4—Fe1—O1	90.57 (11)	C17—C18—H18A	119.9
O5—Fe1—N2	108.53 (12)	C19—C18—H18A	119.9
O2—Fe1—N2	84.74 (11)	C18—C19—C20	120.3 (4)
O4—Fe1—N2	92.83 (11)	C18—C19—H19A	119.9
O1—Fe1—N2	74.54 (11)	C20—C19—H19A	119.9
O5—Fe1—N4	84.04 (11)	C21—C20—C19	119.8 (4)
O2—Fe1—N4	111.65 (11)	C21—C20—H20A	120.1
O4—Fe1—N4	74.63 (11)	C19—C20—H20A	120.1
O1—Fe1—N4	89.13 (11)	C20—C21—C16	119.9 (4)
N2—Fe1—N4	159.46 (12)	C20—C21—C22	118.4 (4)
C2—C1—C6	118.9 (4)	C16—C21—C22	121.7 (4)
C2—C1—H1A	120.5	O4—C22—N3	118.8 (4)
C6—C1—H1A	120.5	O4—C22—C21	120.9 (3)
C3—C2—C1	120.9 (4)	N3—C22—C21	120.3 (3)
C3—C2—H2A	119.6	N4—C23—C24	123.6 (3)
C1—C2—H2A	119.6	N4—C23—H23A	118.2
C4—C3—C2	121.0 (4)	C24—C23—H23A	118.2
C4—C3—H3A	119.5	C25—C24—C29	119.5 (4)
C2—C3—H3A	119.5	C25—C24—C23	122.4 (3)
C5—C4—C3	119.3 (4)	C29—C24—C23	118.1 (3)
C5—C4—H4A	120.3	O5—C25—C24	123.5 (4)
C3—C4—H4A	120.3	O5—C25—C26	118.4 (3)
C4—C5—C6	120.9 (4)	C24—C25—C26	118.1 (3)
C4—C5—H5A	119.5	O6—C26—C27	125.5 (4)
C6—C5—H5A	119.5	O6—C26—C25	114.2 (3)
C5—C6—C1	118.9 (4)	C27—C26—C25	120.3 (4)
C5—C6—C7	118.2 (4)	C26—C27—C28	120.6 (4)
C1—C6—C7	122.8 (4)	C26—C27—H27A	119.7
O1—C7—N1	120.0 (4)	C28—C27—H27A	119.7
O1—C7—C6	120.3 (3)	C29—C28—C27	121.0 (4)
N1—C7—C6	119.8 (4)	C29—C28—H28A	119.5
N2—C8—C9	124.2 (4)	C27—C28—H28A	119.5
N2—C8—H8A	117.9	C28—C29—C24	120.5 (4)
C9—C8—H8A	117.9	C28—C29—H29A	119.8
C10—C9—C14	119.3 (4)	C24—C29—H29A	119.8
C10—C9—C8	123.0 (3)	O6—C30—H30A	109.5
C14—C9—C8	117.7 (4)	O6—C30—H30B	109.5
O2—C10—C9	122.9 (3)	H30A—C30—H30B	109.5
O2—C10—C11	118.8 (4)	O6—C30—H30C	109.5
C9—C10—C11	118.3 (3)	H30A—C30—H30C	109.5
O3—C11—C12	125.1 (4)	H30B—C30—H30C	109.5
O3—C11—C10	114.3 (3)	C7—N1—N2	114.4 (3)
C12—C11—C10	120.6 (4)	C7—N1—H1B	122.8
C13—C12—C11	120.6 (4)	N2—N1—H1B	122.8
C13—C12—H12A	119.7	C8—N2—N1	117.5 (3)
C11—C12—H12A	119.7	C8—N2—Fe1	129.2 (3)
C14—C13—C12	120.3 (4)	N1—N2—Fe1	113.2 (2)
C14—C13—H13A	119.8	C22—N3—N4	115.3 (3)
C12—C13—H13A	119.8	C22—N3—H3B	122.4
C13—C14—C9	120.8 (4)	N4—N3—H3B	122.4
C13—C14—H14A	119.6	C23—N4—N3	117.7 (3)
C9—C14—H14A	119.6	C23—N4—Fe1	129.1 (3)
O3—C15—H15A	109.5	N3—N4—Fe1	112.6 (2)
O3—C15—H15B	109.5	C7—O1—Fe1	117.7 (2)
H15A—C15—H15B	109.5	H1WA—O1W—H1WB	121.9
O3—C15—H15C	109.5	C10—O2—Fe1	135.8 (3)
H15A—C15—H15C	109.5	C11—O3—C15	118.2 (3)
H15B—C15—H15C	109.5	C22—O4—Fe1	118.6 (2)
C17—C16—C21	119.7 (4)	C25—O5—Fe1	135.6 (2)
C17—C16—H16A	120.1	C26—O6—C30	118.0 (3)

### Hydrogen-bond geometry (Å, °)

**Table:**

*D*—H···*A*	*D*—H	H···*A*	*D*···*A*	*D*—H···*A*
N1—H1B···Cl1^i^	0.86	2.25	3.087 (3)	163
N3—H3B···O1W	0.86	1.92	2.759 (4)	164
O1W—H1WA···O5^ii^	0.85	2.39	3.045 (4)	134
O1W—H1WB···Cl1	0.85	2.37	3.198 (3)	163

Symmetry codes: (i) *x*−1, *y*, *z*; (ii) −*x*+1, −*y*+1, −*z*+2.

## References

[bb1] Brandenburg, K. (1999). *DIAMOND* Crystal Impact GbR, Bonn, Germany.

[bb2] Bruker (2007). *SMART* and *SAINT-Plus* Bruker AXS Inc., Madison, Wisconsin, USA.

[bb3] Dilworth, J.-R. (1976). *Coord. Chem. Rev.* **21**, 29–62.

[bb4] Gao, S., Weng, Z.-Q. & Liu, S.-X. (1998). *Polyhedron*, **17**, 3595–3606.

[bb5] Merchant, J. R. & Clothia, D. S. (1970). *J. Med. Chem.* **13**, 335–336. 10.1021/jm00296a058 5418529

[bb6] Monfared, H. H., Sadighian, S., Kamyabi, M. A. & Mayer, P. (2007). *J. Mol. Catal. A*, **304**, 139–146.

[bb7] Pickart, L., Goodwin, W. H., Burgua, W., Murphy, T. B. & Johnson, D. K. (1983). *Biochem. Pharmacol.* **32**, 3868–3871. 10.1016/0006-2952(83)90164-8 6661260

[bb8] Pouralimardan, O., Chamayou, A. C., Janiak, C. & Monfared, H. H. (2007). *Inorg. Chim. Acta*, **360**, 1599–1608.

[bb9] Sacconi, L. (1954). *Z. Anorg. Allg. Chem.* **275**, 249–256.

[bb10] Sheldrick, G. M. (1996). *SADABS* University of Göttingen, Germany.

[bb11] Sheldrick, G. M. (2008). *Acta Cryst.* A**64**, 112–122. 10.1107/S0108767307043930 18156677

[bb12] Westrip, S. P. (2010). *J. Appl. Cryst.* **43** Submitted.

[bb13] Yu, G.-M., Li, Y.-H., Zou, L.-F., Zhu, J.-W. & Liu, X.-Q. (2010). *Acta Cryst.* E**66**, m693–m694. 10.1107/S1600536810018040 PMC2979386 21579331

